# *Streptococcus agalactiae* infective endocarditis complicated by multiple mycotic hepatic aneurysms and massive splenic infarction: a case report

**DOI:** 10.1186/s12876-017-0728-0

**Published:** 2017-12-29

**Authors:** Pietro Achilli, Angelo Guttadauro, Paolo Bonfanti, Sabina Terragni, Luca Fumagalli, Ugo Cioffi, Francesco Gabrielli, Matilde De Simone, Marco Chiarelli

**Affiliations:** 1 University of Milan - Fondazione IRCCS Ca’ Granda Ospedale Maggiore Policlinico, Via Sforza 35, 20122 Milan, Italy; 2 Department of Surgery, University of Milan-Bicocca, Istituti Clinici Zucchi, Via Zucchi, 24, 20900 Monza, Italy; 3 Infectious Diseases Unit - A. Manzoni Hospital, Via dell’Eremo 9/11, 23900 Lecco, Italy; 4 Department of Surgery, Ospedale Alessandro Manzoni, Lecco, Via dell’Eremo 9/11, 23900 Lecco, Italy; 5 Department of Surgery, University of Milan, Milan, Italy

**Keywords:** Hepatic artery aneurysm, Splenic infarction, Infective endocarditis, *Streptococcus agalactiae*

## Abstract

**Background:**

The burden of disease caused by *Streptococcus agalactiae* has increased significantly among older adults in the last decades. Group B streptococcus infection can be associated with invasive disease and severe clinical syndromes, such as meningitis and endocarditis.

**Case presentation:**

We present the case of a 56-year-old man who developed multiple mycotic aneurysms of the right hepatic artery and massive splenic infarction as rare complications of *Streptococcus agalactiae* infective endocarditis. The patient underwent urgent right hepatic artery ligation and splenectomy. The postoperative course was complicated by an episode of hemobilia due to the rupture of a partially thrombosed mycotic aneurysm into the biliary tree. Thus, selective radiological embolization of the left hepatic artery branches was necessary.

**Conclusion:**

To our knowledge, this is the first case reported of infected aneurysms of visceral arteries caused by Group B streptococcus infection. Clinical and laboratory findings were non-specific, while imaging features with computed tomography scan and angiography were highly suggestive. In our case, early recognition, culture-specific intravenous antibiotics and urgent surgical treatment combined with interventional radiology played a decisive role in the final result.

## Background

*Streptococcus agalactiae*, known as Group B streptococcus (GBS), is a Gram-positive coccus commonly associated with infective disease of pregnant women and newborns [1]. Recent large studies suggest an increasing incidence of invasive GBS disease among non-pregnant adults in Western countries [2, 3]. Common presentations of GBS disease in adults include soft-tissue and skin infection, pneumonia, urinary tract infection and severe clinical syndromes, such as meningitis and endocarditis. We present the first case of GBS endocarditis in a healthy adult patient complicated by multiple mycotic aneurysms (MAs) of hepatic artery and massive splenic infarction (SI).

## Case presentation

**Fig. 1:**
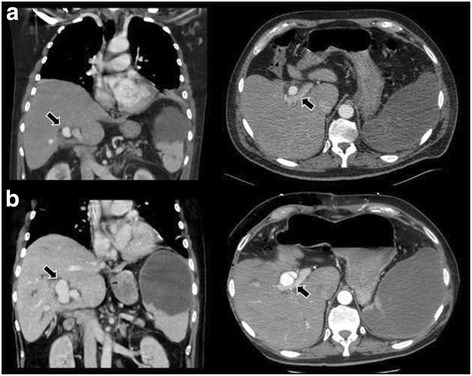
Abdominal CT taken on day 3 (**a**) shows MA of the proximal right hepatic artery of 1.4 cm × 1.1 cm (*arrow*). Moreover the diagnostic imaging discloses a nonenhancing hypodense area of the spleen consistent with SI. Abdominal enhanced CT taken on day 20 (**b**) reveals an enlargement of the previously detected MA of the proximal right hepatic artery (*arrow*) and the development of a new large MA of the VIII segment (*arrow*). The radiological findings confirm SI

**Fig. 2:**
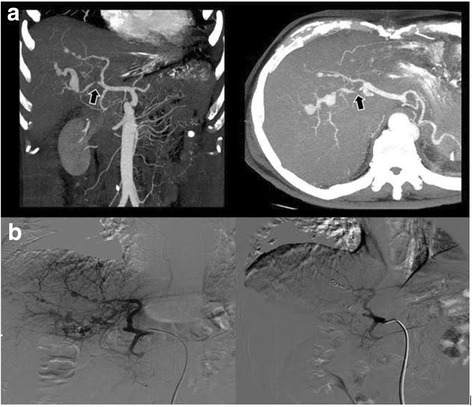
Multislice abdomen CT angiography taken on day 42 (**a**) showing ligation of the right hepatic artery (*arrow*) and three small MAs of the IV hepatic segment. The radiological images demonstrate residual blood flow in the right hepatic MAs. Selective angiography of the coeliac axis (**b**) demonstrates arterial branches from the left hepatic artery providing blood supply to the right hepatic MAs

**Fig. 3:**
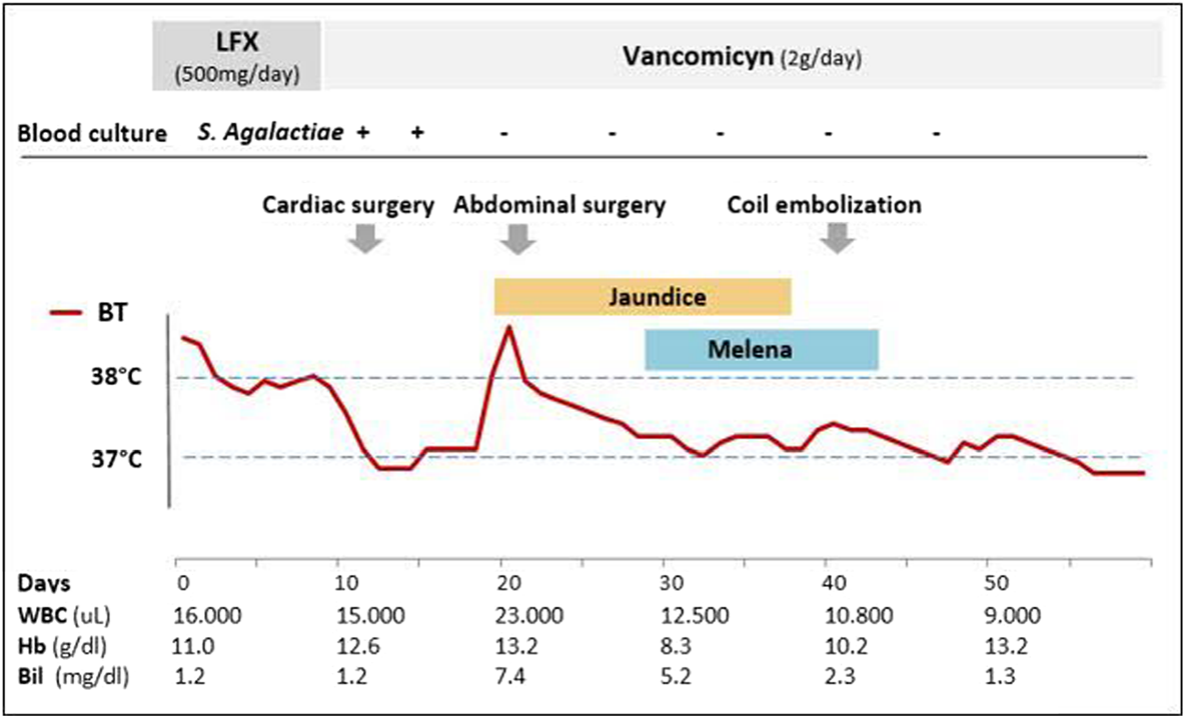
Clinical timeline of the case. BT: body temperature; WBC: white blood cell count; Hb: hemoglobin; Bil: total bilirubin level; LFX: levofloxacin

A 56-year-old man was referred to our emergency department with a 10 days history of pyrexia, productive cough and appetite loss. He reported an adverse reaction to penicillin and he had no history of intravenous drug abuse. Physical examination showed coarse crackles in lower right lung field, and patient’s laboratory workup was remarkable for elevated C-reactive protein (CRP) (16 mg/dl) and leukocytosis (16.000/mm^3^) with neutrophilia of 88%. Chest X-ray showed right lobar consolidation. The patient was admitted to our hospital based on a diagnosis of community-acquired pneumonia. Blood samples for hemocultures were collected and empirical therapy with levofloxacin (500 mg/day) was undertaken (day 1). Considering the worsening of the patient’s general conditions, a computed tomography (CT) scan of abdomen and thorax was performed: the diagnostic imaging revealed left pleural effusion, extensive SI caused by thrombosis of the splenic artery and a hyperdense round lesion of the right hepatic lobe suspected for hepatic aneurysm (1.4 cm × 1.1 cm) (Fig. 1a). Blood cultures, taken on day 1, were positive for penicillin-sensible GBS and the patient immediately underwent a transthoracic and transesophageal echocardiography finding an infective vegetation (1.0 cm × 1.5 cm) on the mitral valve associated with mitral chordae tendineae rupture and severe regurgitation. Intravenous antibiotic therapy was consequently switched to intravenous vancomycin (2 g/day) and emergency mitral valve repair with artificial chordal replacement was performed (day 12). During post-acute cardiac rehabilitation program the patient developed jaundice and laboratory tests showed a total bilirubin of 7.4 mg/dl and elevation of alkaline phosphatase and γ-glutamyl transferase (day 20). A new emergent abdomen CT scan found an enlarging MA of the right hepatic artery of 2.4 cm × 2.0 cm of size and a 2.7 cm × 3.0 cm intrahepatic aneurysm in segment VIII, causing displacement of the portal vein and compression of the right biliary duct (Fig. 1b). Furthermore, CT images detected three smaller left hepatic artery aneurysms located in segment IV and confirmed massive SI. Thus, multiple hepatic MAs associated with GBS endocarditis was diagnosed. The patient was immediately submitted to surgical intervention, which consisted in ligation of the right hepatic artery and splenectomy. Initial postoperative course was favorable with reduction of conjugated bilirubin level. A duplex ultrasonography performed 4 days after surgery demonstrated complete thrombosis of the proximal right hepatic artery aneurysm, partial thrombosis of the aneurysm in segment VIII and no further enlargement of the small aneurysms of the left hepatic artery. However, on day 28, an episode of melena associated with low hemoglobin count emerged. The patient underwent esophagogastroduodenoscopy and colonoscopy, which did not show any gastrointestinal bleeding. On day 42, because of progressive decrease of hemoglobin level, a multislice abdomen CT angiography was performed (Fig. 2a). The radiological findings were consistent with hemobilia due to a suspected rupture of the partially thrombosed MA of segment VIII into the biliary tree. On the following day, the patient underwent a selective angiography of the coeliac axis, which revealed blood supply to the segment VIII MA from intraparenchymal branches of the left hepatic artery. The patient was then successfully treated with transcatheter coil embolization, resulting in complete obliteration of the residual segment VIII MA and of the three segment IV small aneurysms (Fig. 2b). The post-procedural course was uneventful and the patient was discharged on day 58, after 10 weeks of antibiotic therapy. A follow-up CT angiography was performed 8 weeks later, showing complete embolization of the residual MAs without any evidence of bile duct dilatation. The patient remained asymptomatic at follow-up of 12 months, with no signs of relapse of biliary obstruction or gastrointestinal bleeding (Fig. 3).

## Discussion

*Streptococcus agalactiae* has been traditionally recognized as a cause of sepsis and meningitis in newborns and pregnant women [1, 2]. Today, given the decline of neonatal GBS disease in Western countries, as a result of effective intrapartum prophylaxis, the majority of all invasive GBS infections occur in adult people [2, 3]. There are many underlying conditions that can lead to an increased risk for invasive GBS disease, including diabetes, cancer, congestive heart failure, chronic obstructive pulmonary disease and cirrhosis [3, 4]. Clinical manifestations of GBS infection in adults are quite varied [4–6]. Arthritis and upper respiratory tract infections are more frequently reported in adults (< 70 years) while skin infections, pneumonia and urinary tract infection are more common among the elderly (>70 years) [5]. Infective endocarditis (IE) caused by GBS infection is uncommon [4, 5, 7]. It represents one of the most severe presentation of GBS infection and it is associated with a high mortality rate [8]. Regarding antibiotic therapy, the combination of β-lactam antibiotic with aminoglycoside can be considered the treatment of choice [7, 9, 10]. In our case, considering the allergy to penicillin of our patient, we successfully used vancomycin as an alternative to β-lactam antibiotic [7, 9]. The peculiar feature of our case consisted in the development of two simultaneous complications such as SI and multiple MAs of the hepatic artery, as a consequence of septic emboli from valvular vegetations.

SI is a relatively uncommon diagnosis. Infarction of the spleen is usually due to the occlusion of the splenic artery secondary to thrombosis or embolism. SI causes include hematologic diseases (sickle cell disease, polycythemia vera), hypercoagulability disorders (antiphospholipid syndrome, cancer) and embolic events (atrial fibrillation, infective endocarditis) [11, 12]. Complications include splenic abscess, pseudocysts and hemorrhage [13]. Clinical presentation of SI consists in left upper quadrant pain irradiated to the shoulder, often associated with anemia and leukocytosis; fever, splenomegaly and left pleural effusion are common findings in SI [11–13]. Lactate dehydrogenase serum levels are frequently elevated [11, 12]. The most sensitive imaging modality is the contrast-enhanced CT, which can show wedged shape hypodense areas in the spleen, splenomegaly with perisplenic fluids and left-sided pleural effusion [11]. When SI is diagnosed, all possible etiologies should be considered and a scrutiny on the source of emboli should be carried out. Intravenous antibiotic therapy based on cultures results is the mainstay of the treatment [11, 12]. Splenectomy is rarely required and surgery is usually considered in case of complications or when medical treatment is ineffective [11, 13]. Unusually our patient did not report any abdominal tenderness or pain at time of diagnosis, while anemia, leukocytosis and pleural effusion were present. In our report the indication for splenectomy was the persistence of septic condition and the need of urgent surgery for hepatic aneurysm.

MA is a rare disease characterized by elusive clinical presentation and high mortality rate [14]. The diffusion of antibiotic therapy in the clinical practice has reduced the incidence of MAs: actually infected aneurysms comprise 1-2.5% of all aortic aneurysms [15]. The typical feature of this pathologic entity is the destruction of the arterial wall caused by bacterial infection leading to vessel dilatation. The etiology of MA is mainly ascribed to septic embolism of vasa vasorum originating from valvular vegetations in the setting of infective endocarditis; direct colonization of damaged atherosclerotic arterial wall during bacteremia is considered a rare pathogenetic mechanism [16]. Most frequently, MAs occur in abdominal aorta, peripheral arteries, especially femoral artery, and cerebral arteries. Infected aneurysms of visceral arteries are relatively infrequent and generally show an aggressive clinical course with significant mortality [14]. Multidetector CT angiography is considered the pivotal imaging modality in the characterization of MA, showing contrast-enhancing saccular dilatation of the artery with central or eccentric lumen [14]. The most frequent infective agents related to the development of these aneurysms in post-antibiotic era are *Staphylococcus aureus* and Salmonella spp.; β-hemolytic group A Streptococci, Streptococcus pneumoniae and Haemophilus influenzae were more common in the past decades [14, 16]. GBS infection rarely causes MAs and only seven other cases are described in the literature: six of them involving the thoracic or abdominal aorta and one the femoral artery. To our knowledge, this is the first case reported of mycotic aneurysm of visceral arteries caused by *Streptococcus agalactiae*. Hepatic artery MA is usually asymptomatic, but in a minority of cases right upper quadrant pain, jaundice and digestive bleeding may be present [16]. The management of infected aneurysms of hepatic arteries consists in surgical treatment or in endovascular embolization [17]. Although only small series are published, urgent treatment (surgical or radiologic) is generally recommended in symptomatic patients or when aneurysm is larger than 2 cm: these conditions are associated with a higher risk of rupture than atherosclerotic aneurysms [17]. Surgical option is preferable in patients with low surgical risk and proximal aneurysms; embolization should be considered in case of intrahepatic lesions or in high-risk surgical candidates [16]. Antibiotics, possibly tailored on culture sensitivity, are invariably indicated and should be continued for at least 6-8 weeks postoperatively [14]. In our case, we opted for urgent surgical intervention considering the high risk of rupture of the proximal right hepatic aneurysm, proved by its rapid enlargement (from 1.4 to 2.4 cm within 17 days), the sudden increase of bilirubin level and the development of new lesions. In addition, a concomitant splenectomy was required and consequently the open procedure seemed to be the best choice. Unfortunately, the surgical ligation of the right hepatic artery did not result in a complete obliteration of the aneurysms and subsequently endovascular embolization became necessary. This was due to collateral arterial branches from the left hepatic artery that provided blood supply to the right hepatic aneurysms. After selective radiological embolization of the left hepatic artery branches of IV segment, anemia and jaundice were resolved.

## Conclusion

We reported the first case of multiple hepatic MAs associated with massive SI, caused by GBS infection in a healthy adult patient. While clinical and laboratory findings were not specific, imaging features with CT scan and angiography were highly suggestive. In our case, early recognition, culture-specific intravenous antibiotics and urgent surgical treatment combined with interventional radiology were crucial factors to the final result.

## Abbreviations

CRP: C-reactive protein
CT: Computed tomography
GBS: Group B streptococcus
IE: Infective endocarditis
MA: Mycotic aneurysm
SI: Splenic infarction
